# Associations between Prenatal and Postnatal Exposure to Cannabis with Cognition and Behavior at Age 5 Years: The Healthy Start Study

**DOI:** 10.3390/ijerph20064880

**Published:** 2023-03-10

**Authors:** Brianna F. Moore, Kaytlyn A. Salmons, Adrienne T. Hoyt, Karli S. Swenson, Emily A. Bates, Katherine A. Sauder, Allison L. B. Shapiro, Greta Wilkening, Gregory L. Kinney, Andreas M. Neophytou, Cristina Sempio, Jost Klawitter, Uwe Christians, Dana Dabelea

**Affiliations:** 1 Department of Epidemiology, Human Genetics and Environmental Sciences, Health Science Center, The University of Texas, Austin, TX 78712, USA; 2 Lifecourse Epidemiology of Adiposity and Diabetes (LEAD) Center, Colorado School of Public Health, Aurora, CO 80045, USA; 3 Department of Epidemiology, Colorado School of Public Health, Aurora, CO 80045, USA; 4 Department of Environmental and Radiological Health Sciences, Colorado State University, Fort Collins, CO 80521, USA; 5 Department of Health Promotion and Behavioral Science, Health Science Center, The University of Texas, Austin, TX 78712, USA; 6 Department of Pediatrics, School of Medicine, University of Colorado, Aurora, CO 80045, USA; 7 Department of Anesthesiology, School of Medicine, University of Colorado, Aurora, CO 80045, USA

**Keywords:** cannabis, cannabidiol, delta-9-tetrahydrocannabinol, behavior, cognition, aggression, attention deficit and disruptive behavior disorders, language development

## Abstract

Background: Prenatal exposure to cannabis may influence childhood cognition and behavior, but the epidemiologic evidence is mixed. Even less is known about the potential impact of secondhand exposure to cannabis during early childhood. Objective: This study sought to assess whether prenatal and/or postnatal exposure to cannabis was associated with childhood cognition and behavior. Study design: This sub-study included a convenience sample of 81 mother–child pairs from a Colorado-based cohort. Seven common cannabinoids (including delta 9-tetrahydrocannabinol (Δ9-THC) and cannabidiol (CBD)) and their metabolites were measured in maternal urine collected mid-gestation and child urine collected at age 5 years. Prenatal and postnatal exposure to cannabis was dichotomized as exposed (detection of any cannabinoid) and not exposed. Generalized linear models examined the associations between prenatal or postnatal exposure to cannabis with the NIH Toolbox and Child Behavior Checklist T-scores at age 5 years. Results: In this study, 7% (*n* = 6) of the children had prenatal exposure to cannabis and 12% (*n* = 10) had postnatal exposure to cannabis, with two children experiencing this exposure at both time points. The most common cannabinoid detected in pregnancy was Δ9-THC, whereas the most common cannabinoid detected in childhood was CBD. Postnatal exposure to cannabis was associated with more aggressive behavior (β: 3.2; 95% CI: 0.5, 5.9), attention deficit/hyperactivity problems (β: 8.0; 95% CI: 2.2, 13.7), and oppositional/defiant behaviors (β: 3.2; 95% CI: 0.2, 6.3), as well as less cognitive flexibility (β: −15.6; 95% CI: −30.0, −1.2) and weaker receptive language (β: −9.7; 95% CI: −19.2, −0.3). By contrast, prenatal exposure to cannabis was associated with fewer internalizing behaviors (mean difference: −10.2; 95% CI: −20.3, −0.2) and fewer somatic complaints (mean difference: −5.2, 95% CI: −9.8, −0.6). Conclusions: Our study suggests that postnatal exposure to cannabis is associated with more behavioral and cognitive problems among 5-year-old children, independent of prenatal and postnatal exposure to tobacco. The potential risks of cannabis use (including smoking and vaping) during pregnancy and around young children should be more widely communicated to parents.

## 1. Introduction

Cannabis use is becoming increasingly common among pregnant people and parents. In 2017, 7% of pregnant people and 11% of U.S. adults with children in the home reported past-month use [1,2]. Yet, these self-reported measures may be an underestimation of the actual prevalence of this exposure [3]. Convenience samples in urban medical centers across the United States reported that one in four pregnant patients [4,5] and one in five child patients, between the ages of 0 and 3 years [6,7], tested positive for cannabis.

The relatively high prevalence of this exposure is a concern because early-life exposures to cannabis may alter the child’s cognitive and behavioral development. At least 44 epidemiologic reports have examined the potential impact of prenatal exposure to cannabis on cognitive outcomes among offspring, ages 1–22 years. Fifteen of these reports found strong evidence of an association between prenatal exposure to cannabis and impaired cognition, including lower developmental quotients [8] and general intelligence scores [9,10,11]; impaired executive functioning [12,13,14], language development [15,16], and memory [10,17,18]; slower processing speed [19]; poorer academic performance [20,21]; and lower cognition scores [22]. Conversely, five of the reports found evidence that prenatal exposure to cannabis was associated with some improvements in cognition, including improved cognition scores [23], comprehension [24], motor control [25,26], and academic performance [27]. An additional 24 reports found limited effects of prenatal exposure to cannabis on child cognition [28,29,30,31,32,33,34,35,36,37,38,39,40,41,42,43,44,45,46,47,48,49,50,51].

Fewer studies have assessed whether prenatal exposure to cannabis is associated with childhood behavior. To our knowledge, 18 epidemiologic reports have examined the association between prenatal exposure to cannabis and offspring behavior between 0–16 years of age [22,23,24,51,52,53,54,55,56,57,58,59,60,61,62,63,64,65,66]. Most reports found evidence that prenatal exposure to cannabis was associated with greater presentation of externalizing behaviors [22,55,63], aggressive behaviors [52,63,64], attention problems [22,51,52,53,54,55,64,65], depressive symptoms [56,57,64], and other behavioral changes [55,62,64] in the offspring, while others reported no association between prenatal exposure to cannabis and offspring behavior [23,58,59,60,61,66]. Two reports suggested that cannabis-exposed offspring may exhibit improved sustained attention [24,60].

There is a paucity of data on the potential impact of postnatal exposure to cannabis on child cognition and behavior. In a diverse cohort of women with low household incomes and low maternal education, Eiden and colleagues [67] reported that maternal cannabis use during the first year of life was associated with behavioral problems at age 2 and 3 years. More recently, Wade and colleagues [68] observed that postnatal exposure to cannabis was associated with poorer memory but better performance on tests of oral reading among children, ages 10–13 years, in the Adolescent Brain Cognitive Development (ABCD) Study.

To address these gaps in knowledge, we leveraged data from an ongoing, Colorado-based pre-birth cohort. We sought to estimate the associations between prenatal and postnatal exposure to cannabis with childhood cognition and behavior at age 5 years. A novel aspect of our approach is the objective measurement of twelve cannabinoids/metabolites in both maternal urine (collected mid-gestation) and child urine (collected at age 5 years). We hypothesized that both prenatal and postnatal exposure to cannabis would be associated with more cognitive and behavioral problems at age 5 years, independent of early-life exposure to tobacco and other sociodemographic factors.

## 2. Methods

Healthy Start is a racially and ethnically diverse cohort of 1410 mothers and their offspring born between 2010 and 2014. Pregnant females were recruited from the outpatient obstetrics clinics at the University of Colorado Hospital prior to 24 weeks of gestation. Participants were excluded from this study if they were expecting multiple births or had pre-existing diabetes, asthma, cancer, or a self-reported psychiatric illness. Enrolled pregnant people were invited to participate in three pregnancy visits at <23 weeks gestation, 24–28 weeks gestation, and delivery. A childhood follow-up visit occurred in-person when children were 5 years of age, between 2015 to 2021. Prior to participation, written informed consent was obtained. The protocol was approved by the Colorado Multiple Institution Review Board.

Mother–child pairs were eligible for the current analysis if they had complete exposure data (cannabinoids were measured in stored child urine samples) and complete outcome data (the mother completed the Child Behavior Checklist (CBCL) and/or the child completed the NIH Toolbox Cognition Battery assessment). Mother–child pairs were excluded from this analysis if delivery occurred prior to 37 weeks of gestation or if they had a birth weight less than 2500 g. These factors are independently linked to neurodevelopmental delays [69], though they are likely to be biological intermediates, rather than confounders [70]. As such, adjusting for these variables would introduce bias [71]. Therefore, we excluded mother–child pairs based on this criterion to understand the impact of cannabis exposure on child cognition and behavior among offspring born full term and at a normal birth weight.

Fetal and postnatal exposure to cannabis: Thirteen cannabinoids/metabolites were measured in stored maternal urine samples collected at 27 weeks gestation and child urine samples collected at age 5 years using a validated high-performance liquid chromatography-tandem mass spectrometry (LC-MS/MS) assay [72]. Samples were stored in polypropylene cryovials at −80 °C until analysis in 2021 (maternal samples) and 2022 (child samples).

In brief, samples were analyzed on an Agilent 1 limited by the cannabis exposure assessment. High-performance liquid chromatography system (Agilent Technologies, Santa Clara, CA, USA) and AB SCIEX API5000 tandem mass spectrometer (Sciex, Concord, ON, Canada) were used, as previously described [72]. Detection was performed in positive atmospheric pressure chemical ionization mode. The analytes measured were as follows: Δ9- tetrahydrocannabinol (THC), 11-hydroxy-Δ9-tetrahydrocannabinol (11OH- Δ9-THC), 11-nor-delta 9-carboxy-tetrahydrocannabinol (Δ9-THC-COOH), Δ9-tetrahydrocannabinol-9-carboxylic acid glucuronide (Δ9-THC-C-gluc), Δ9-tetrahydrocannabinol glucuronide (Δ9-THC-gluc), cannabidiol (CBD), cannabidiol glucuronide (CBD-gluc), 7-carboxy cannabidiol (CBD-COOH), cannabichromene (CBC), cannabinol (CBN), cannabigerol (CBG), Δ9-tetrahydrocannabivarin (THCV), and cannabidivarin (CBDV).

The limit of quantification (LOQ) varied as follows: 0.39 ng/mL (for Δ9-THC), 0.78 ng/mL (for THC-COOH, CBD, CBC, CBG, THCV, and CBDV), 1.56 ng/mL (for 11OH- Δ9-THC, THC-gluc, CBD-gluc, and CBN), and 7.82 ng/mL (for Δ9-THC-C-gluc). Due to the limited sample size, postnatal exposure to cannabis was dichotomized as exposed (where any cannabinoid/metabolite was at or above the lower LOQ) and not exposed (where no cannabinoid or cannabinoid metabolites reached the lower LOQ). For the prenatal exposure to cannabis variable, we dichotomized the variable based on the limit of detection (LOD), which is approximately half of the LOQ. This is in line with the Clinical Laboratory Institute guidelines, which allow for the use of concentrations above the LOD and below the LOQ. The rationale for including values below the LOQ and above the LOD was to the ensure that we had a sufficient sample size to assess these associations in this small pilot study.

Childhood cognition: The NIH Toolbox Cognition Battery is a series of tests designed to measure cognitive processes across the lifespan (ages 3 to 85 years) [73]. Three tests in the Cognition Battery were completed by our study population: the Flanker test (inhibitory control) [73], the Dimensional Change Card Sort test (DCCS; cognitive flexibility) [74], and the Picture Vocabulary test (receptive language). During the in-person research visit, children completed the tests on a tablet computer while a trained professional research assistant supervised. Raw scores were based on accuracy and response time (Flanker and DCCS) or accuracy (Picture Vocabulary). T-scores were generated from the raw scores, which are fully corrected for age, sex, race (black, other, white), ethnicity (Hispanic vs. non-Hispanic), and mother’s educational attainment [75]. The mean fully corrected T-score range for all tests is 50, with a standard deviation (SD) of 10 [75].

Childhood behavior: The preschool version of the Child Behavior Checklist (CBCL) was completed by mothers at the 5-year research visit. The CBCL is a widely used assessment of behavioral adaption with excellent reliability and validity [76]. Mothers responded to a series of 100 questions regarding their child’s behavior over the previous two months. Each item was scored 0 (not true), 1 (somewhat or sometimes true), or 2 (very true or often true). Raw scores were used to calculate T-scores, based upon age and sex of the child. The T-scores have a normal distribution with a mean of 50 and a standard deviation of 10. The threshold for subclinical range is T-scores ≥ 60. T-scores are used to construct three broadband scales, seven syndrome scales, and five Diagnostic and Statistical Manual of Mental Disorders (DSM)-oriented scales. The total problems T-score is the sum score of the 100 problem items. The internalizing problems T-score is the sum score of four syndrome scales (emotionally reactive, anxious/depressed, somatic complaints, and withdrawn). The externalizing problems T-score is the sum score of two syndrome scales (attention problems and aggressive behavior). The five DSM-oriented scales include depressive problems, anxiety problems, attention deficit/hyperactivity problems, autism spectrum problems, and oppositional/defiant problems).

Covariates: Cotinine (the major metabolite of nicotine [77]) was measured in maternal urine samples collected at 27 weeks gestation and child urine samples collected at age 5 years. Cotinine was measured via solid-phase competitive ELISA, with a sensitivity of 1 ng/mL (Calbiotech Cotinine ELISA CO096D). The LOD was 0.05 ng/mL. Prenatal or childhood exposure to tobacco was defined as follows: any exposure (cotinine ≥ LOD, indicating maternal active cigarette/e-cigarette use or exposure to secondhand smoke) and no exposure (cotinine < LOD).

Maternal age at delivery was calculated based on offspring delivery date and maternal date of birth. Maternal education, race, ethnicity, and annual household income were collected via questionnaires. Maternal height was measured using a stadiometer at the first prenatal research visit. Pre-pregnancy weight was obtained from medical records or from questionnaires completed at enrollment. Gestational weight gain was calculated as the difference between the last available weight measurement during pregnancy and the pre-pregnancy weight. Although maternal psychiatric illness was part of the initial exclusion criteria, some participants did not self-report a condition or were diagnosed after recruitment into our study. Therefore, we obtained information about maternal psychiatric disorders (non-specified) via medical records.

Statistical analyses: Generalized linear models estimated the associations between prenatal or postnatal exposure to cannabis (not exposed or exposed) with age- and sex-corrected behavior and fully corrected cognition T-scores at age 5 years. We present adjusted beta coefficients with corresponding 95% confidence intervals (CIs) for all models. Our models adjusted for potential confounders and precision variables, which were identified by the construction of a directed acyclic graph. These covariates include maternal age (years), maternal education (college degree or less than a college degree), maternal race and ethnicity (Hispanic, non-Hispanic black, non-Hispanic other, and non-Hispanic white), a non-specified maternal psychiatric diagnosis (yes or no), child sex, child age at the behavioral or cognitive assessment (years), prenatal exposure to tobacco (maternal cotinine < LOD or maternal cotinine ≥ LOD), and childhood exposure to tobacco (child cotinine < LOD or child cotinine ≥ LOD).

Stata version 14.2 (StataCorp LP, College Station, TX, USA) was used for all analyses. The criterion for significance was set at *p* < 0.05.

## 3. Results

Healthy Start initially enrolled 1410 participants. A subsample of 199 participants with stored urine samples collected at 27 weeks gestation were included in this sub-study. Of these, twenty participants were excluded due to delivery prior to 37 weeks (*n* = 11) or a birth weight less than 2500 g (*n* = 9). An additional 86 participants were missing information about maternal psychiatric disorders (*n* = 12) and childhood exposure to tobacco (*n* = 74). Of the eligible sample (*n* = 93), twelve did not complete the CBCL. Therefore, the final analytic sample for the CBCL analyses was 81. Of the eligible sample (*n* = 93), 47 did not complete the NIH Toolbox Cognition Battery, since this assessment was introduced later during the study. Therefore, the final analytic sample for the NIH Toolbox analyses was 42.

Compared to the full sub-study (*n* = 199), participants in the analytic sample (*n* = 81) were less likely to have prenatal exposure to cannabis (prevalence of 13% and 7%, respectively) and postnatal exposure to cannabis (prevalence of 19% and 12%) (Supplemental Table S1). Compared to all participants initially enrolled in the Healthy Start cohort (*n* = 1410), participants in the sub-study (*n* = 199) and the analytic sample (*n* = 81) were more educated, had higher incomes, were less likely to have a maternal diagnosis of a psychiatric illness, and had offspring with higher birthweights. Differences in maternal age, pre-pregnancy BMI, gestational weight gain, infant sex, or gestational age at birth between the sub-study and analytical sample were negligible.

Table 1 shows characteristics of the 81 mother–child pairs in this study by prenatal and postnatal exposure to cannabis. Some maternal characteristics were discordant across the prenatal and postnatal exposure to cannabis categories. Specifically, mothers whose offspring showed prenatal exposure to cannabis were less educated, had lower household incomes, and were less likely to identify as Hispanic or non-Hispanic white, whereas mothers whose offspring showed postnatal exposure to cannabis were more educated, had higher household incomes, and were more likely to identify as Hispanic. Mothers whose children showed fetal or postnatal exposure to cannabis were more likely to have diagnosed psychiatric disorders and to be younger. Offspring with prenatal or postnatal exposure to cannabis were more likely to be female, had lower birthweights, and were less likely to exclusively breastfeed for 5 months. Offspring with prenatal exposure to cannabis were born to younger mothers. There was no difference in pre-pregnancy BMI, gestational weight gain, or gestational age at birth across the prenatal or postnatal exposure to cannabis categories.

Cannabinoids were detected in 7% of the maternal urine samples and 12% of the child urine samples (Table 2). In maternal urine, the most commonly detected cannabinoid metabolite was Δ9-THC-C-gluc (*n* = 6, 7%), followed by Δ9-THC-COOH (*n* = 4, 5%), and Δ9-THC-gluc (*n* = 4, 5%). Of those with detectable levels of CBD or CBG in maternal urine, Δ9-THC-C-gluc was also detected in urine. In child urine, the most commonly detected cannabinoid metabolite was CBD-gluc (*n* = 9, 11%), followed by CBD-COOH (*n* = 1, 1%). None of the children had detectable levels of the following cannabinoid metabolites in urine: Δ9-THC, Δ9-THC-COOH, Δ9-THC-gluc, 11OH-Δ9-THC-gluc, CBC, CBN, CBG, THCV, or CBDV.

Table 3 compares prenatal and childhood exposures to tobacco and cannabis. There was some discordance between the measures. For instance, among those with prenatal exposure to cannabis (*n* = 6), only two (33%) offspring had postnatal exposure to cannabis, three (50%) had concurrent prenatal exposure to tobacco, and three (50%) had childhood exposure to tobacco. A similar pattern held for postnatal exposure to cannabis. Among those with postnatal exposure to cannabis (*n* = 10), three (30%) offspring had prenatal exposure to tobacco and four (40%) had concurrent childhood exposure to tobacco.

Table 4 shows the associations between early-life exposure to cannabis and child behavior. Postnatal exposure to cannabis was associated with more aggressive behavior (mean difference: 3.2; 95% CI: 0.5, 5.9), attention deficit/hyperactivity problems (mean difference: 8.0; 95% CI: 2.2, 13.7), and more oppositional/defiant behaviors (mean difference: 3.2; 95% CI: 0.2, 6.3) (adjusted for maternal age, maternal education, maternal race and ethnicity, offspring sex, a non-specified maternal psychiatric diagnosis, prenatal exposure to tobacco, childhood exposure to tobacco, and child age at behavioral assessment). On the other hand, prenatal exposure to cannabis was associated with fewer internalizing behaviors (mean difference: −10.2; 95% CI: −20.3, −0.2) and fewer somatic complaints (mean difference: −5.2, 95% CI: −9.8, −0.6).

Table 5 shows the associations between early-life exposure to cannabis and child cognition. Postnatal exposure to cannabis was associated with less cognitive flexibility (mean difference: −15.6; 95% CI: −30.0, −1.2) and less receptive language (mean difference −9.7; 95% CI: −19.2, −0.3). There was no difference in the cognitive measures among those with and without prenatal exposure to cannabis.

## 4. Discussion

Early-life exposure to cannabis is an emerging concern. In this sub-study from a well-phenotyped, pre-birth cohort in Colorado, we observed that 7% (*n* = 6) of the children had prenatal exposure to cannabis and 12% (*n* = 10) of the children had postnatal exposure to cannabis at age 5 years. Additionally, we provide novel evidence that postnatal exposure to cannabis is associated with more aggressive behaviors, attention deficit/hyperactivity problems, and oppositional/defiant behaviors in the offspring, as well as less cognitive flexibility and receptive language, independent of prenatal and childhood exposure to tobacco. These effects were meaningful, with at least one standard deviation difference in behavior and cognition scores between those with and without postnatal exposure to cannabis. Contrary to our hypothesis, prenatal exposure to cannabis was associated with fewer internalizing behaviors and somatic complaints, but not with any of the cognitive outcomes measured.

Our results suggest that postnatal exposure to cannabis is associated with more behavioral issues and poorer cognitive outcomes measured at age 5 years. Certain biological mechanisms could explain the association, such as the role of cannabis in altered expression of glutamatergic neurotransmitters in the cortex [78] or dysregulation of the developing endocannabinoid system [79]. Furthermore, children may be particularly susceptible to secondhand or thirdhand exposures to cannabis, due to their immature detoxification pathways [80], and especially to ambient exposures to cannabis, due to their faster ventilation rates [81]. However, we must be cautious in the interpretation of our findings because the exposure (postnatal exposure to cannabis) and the outcomes (childhood behavior and cognition) were measured at the same time (age 5 years). Thus, it is possible that the exposure occurred because of the outcome. This hypothesis is supported by the work of Eiden and colleagues [67], who reported that maternal cannabis use during the first year of the child’s life was associated with more behavior problems at age 2 years, which in turn predicted cannabis use a year later [67]. In this scenario, cannabis use among parents may be interpreted as a coping strategy in response to their child’s behavior. Further follow-up is needed to understand whether exposures in early childhood are prospectively associated with cognitive and behavioral problems in middle childhood and adolescence, a time when brain structure and function undergo considerable change [82].

We had hypothesized that prenatal exposure to cannabis would be associated with adverse cognitive and behavioral traits in the offspring. This hypothesis is consistent with studies in Wistar rats linking prenatal exposure to Δ9-THC with hyperactivity [83], emotional reactivity [84], and anxiety [84], as well as a recent neuroimaging study reporting disrupted connectivity of brain networks associated with attentional control among cannabis-exposed offspring [85]. Yet, the epidemiologic literature is somewhat mixed, as noted by several reviews [86,87,88], and summarized in Supplemental Tables S2 (for cognitive outcomes) and S3 (for behavioral outcomes). Twenty-four (of forty-four) epidemiologic reports found limited effects on child cognition [28,29,30,31,32,33,34,35,36,37,38,39,40,41,42,43,44,45,46,47,48,49,50,51], and six (of seventeen) epidemiologic reports found limited effects on child behavior [23,58,59,60,61,66]. It is important to note that 14 of the reports showing limited effects on child cognition arose from studies designed to understand the health impacts of heavy cocaine use (rather than cannabis use) during pregnancy [27,28,29,30,35,36,37,38,40,41,42,44,45,46], though 3 reports from these studies reported poorer cognitive outcomes among cannabis-exposed offspring [13,18,19]. An additional eight of the reports showing limited effects on cognition arose from the Maternal Health Practices and Child Development (MHPCD) and the Ottawa Prenatal Prospective Study (OPPS) studies [31,33,34,43,47,48,49,50], which recruited pregnant people between 1978 and 1986. At that time, cannabis was much less potent [89] and may have had more subtle effects on child cognition. Furthermore, exposure assessment varied considerably, with few studies incorporating biomarkers to characterize exposure. This may have biased the published results towards the null, if cannabis use/exposure was under-reported more often among those with child experiencing cognitive and behavioral problems. On the other hand, many reports did not adjust for prenatal exposure to tobacco, an important covariate that is strongly associated with both the exposure [90] and various cognitive [91,92] and behavioral [93,94,95,96,97,98] problems in the offspring, which may result in a bias away from the null.

Contrary to our hypothesis, we found some evidence that prenatal exposure to cannabis was slightly protective against internalizing behaviors and somatic complaints. This could be explained by several of our study’s limitations. First, our results may have been biased by self-selection bias. In our sub-study, we measured cannabinoids in maternal urine among 199 participants. Of these, 13% had detectable cannabinoids in maternal urine collected in mid-pregnancy. Yet, in our analytic study (*n* = 81), only 7% had evidence of prenatal exposure to cannabis. If the loss to follow-up was non-differential with respect to the exposure, this may result in a bias towards the null or, at worst, a change in the direction of the association [99].

Second, our characterization of early-life exposure to cannabis is somewhat limited. Cannabinoids were measured at only one time point during pregnancy and in early childhood. The cannabinoid Δ9-THC has a relatively short half-life (ranging from 20 h to 10 days [100]). As such, our cannabinoid assessment captured only recent exposure and does not reflect exposure over the entire pregnancy or throughout childhood. We also lacked data on self-report of the frequency, mode, or duration of cannabis use, which may influence these associations. Third, while we adjusted for many important covariates, there are other important socioeconomic predictors of neurodevelopment (such as characteristics of the home environment) that we did not measure. Finally, we excluded those with preterm delivery or a low birth weight. Including high-risk offspring may explain some of the findings in the previously published studies.

Consistent with previous research [2], young mothers were more likely to have detectable cannabinoids in urine. Cannabis may be used to treat pregnancy-related symptoms or as an alternative to anti-nausea prescription medications [101]. However, less than 0.5% of cannabis use during pregnancy is strictly for medical purposes [2]. More research is needed to examine reasons for cannabis use in this population.

A distinct advantage of our approach is the population we studied. Pregnant people in Colorado were recruited between 2010 and 2014, amid state-wide legalization of cannabis for recreational use (enacted on 6 November 2012, with retail sales beginning on 1 January 2014), but prior to widespread health messaging about the potential harm to the fetus. This may explain the relatively high prevalence of this exposure (7%) during mid-gestation (after knowledge of the pregnancy) and in early childhood (13%). Additionally, our study is strengthened by the ability to control for many important covariates, including prenatal and childhood exposure to tobacco (confirmed by the measurement of cotinine).

## 5. Conclusions

Pregnant people and parents may use cannabis for recreational or medical purposes. However, they may be unaware of the potential risks to their child. Our study suggests that postnatal exposure to cannabis is associated with more behavioral and cognitive problems among 5-year-old children. However, the association between prenatal exposure to cannabis with childhood cognition and behavior requires further investigation. Nevertheless, it is crucial to educate parents and parents-to-be about the potential risks of cannabis use (including smoking and vaping) during pregnancy and around young children.

## Figures and Tables

**Table 1:** Characteristics of mother–child pairs by prenatal or childhood exposure to cannabis.

		Prenatal Exposure to Cannabis ^a^			Childhood Exposure to Cannabis ^b^		
	All Participants (*n* = 81)	Not Exposed (*n* = 75)	Exposed (*n* = 6)	*p*-Value	Not Exposed (*n* = 71)	Exposed (*n* = 10)	*p*-Value
Mother characteristics							
Age (years)	30 ± 6	31 ± 6	26 ± 6	0.03	30 ± 6	29 ± 5	0.25
Pre-pregnancy BMI (kg/m^2^)	26 ± 5	26 ± 6	27 ± 4	0.33	26 ± 5	27 ± 7	0.79
Gestational weight gain (kg)	13 ± 6	13 ± 6	16 ± 8	0.13	14 ± 5	12 ± 10	0.15
Maternal race and ethnicity							
Non-Hispanic white	59%	61%	33%	<0.01	61%	50%	0.49
Non-Hispanic black	4%	1%	33%		3%	10%	
Hispanic	31%	32%	17%		30%	40%	
Other	6%	5%	17%		7%	0%	
Highest level of education							
<High school	12%	11%	33%	0.24	13%	10%	0.37
High school degree	14%	13%	17%		15%	0%	
Some college or more	74%	76%	50%		72%	90%	
Household income							
<USD 40,000	22%	20%	50%	0.27	20%	20%	0.20
USD 40,001 to USD 70,000	11%	11%	17%		10%	20%	
≥USD 70,000	51%	53%	17%		55%	40%	
Do not know	16%	16%	17%		15%	20%	
Maternal diagnosis of psychiatric illness							
Yes	7%	8%	17%	0.47	7%	20%	0.17
No	91%	92%	83%		93%	80%	
Diet quality during pregnancy (Healthy Eating Index)	63 ± 10	64 ± 10	62 ± 9	0.33	64 ± 10	62 ± 11	0.37
Child characteristics							
Male	48%	51%	17%	0.11	51%	30%	0.22
Birthweight (grams)	3319 ± 383	3338 ± 386	3088 ± 274	0.06	3337 ± 388	3196 ± 338	0.14
Gestational age (weeks)	40 ± 1	40 ± 1	40 ± 1	0.99	40 ± 1	39 ± 1	0.17
Exclusively breastfed at age 5 months							
Yes	57%	59%	40%	0.41	59%	50%	0.60
No	42%	41%	60%		41%	50%	

^a^ Prenatal exposure to cannabis was determined by the detection of twelve cannabinoids/metabolites of cannabis in maternal urine collected at ~27 weeks gestation. The categories of were as follows: exposed (any of the measured cannabinoids exceeded the limit of detection (LOD)) and not exposed (all of cannabinoids measured were below the LOD). ^b^ Childhood exposure to cannabis was determined by the detection of twelve cannabinoids/metabolites of cannabis in child urine collected at age 5 years. The categories of were as follows: exposed (any of the measured cannabinoids exceeded the limit of quantification [LOQ]) and not exposed (all of cannabinoids measured were below the LOQ).

**Table 2:** Cannabinoids/metabolites detected in maternal and child urine samples.

Cannabinoid/ Metabolite	Brief Description	Cannabinoid Detected in Maternal Urine					Cannabinoids Detected in Child Urine				
		LOQ ng/mL	*n* (%)	Mean ± SD	Min	Max	LOD ng/mL	*n* (%)	Mean ± SD	Min	Max
Δ9-THC	Most abundant cannabinoid	0.2	3 (4%)	0.3 ± 0.1	0.3	0.4	0.4	0	-	-	-
11OH-Δ9-THC	Primary metabolite of Δ9-THC	0.8	0				1.6	0	-	-	-
Δ9-THC-COOH	Secondary metabolite of Δ9-THC	0.4	4 (5%)	6.0 ± 7.8	0.7	17.6	0.8	0	-	-	-
Δ9-THC-C-gluc	Glucuronidated Δ9-THC-COOH	3.9	6 (7%)	330 ± 300	100	669	7.8	0	-	-	-
Δ9-THC-gluc	Glucuronidated Δ9-THC	0.8	4 (5%)	4.4 ± 5.2	0.8	12.0	1.6	0	-	-	-
CBD	Second most abundant cannabinoid	0.4	3 (4%)	0.5 ± 0.1	0.5	0.6	0.8	0	-	-	-
CBD-COOH	Secondary metabolite of CBD	0.8	0	-	-	-	1.6	1 (1%)	1.9		
CBD-gluc	Glucuronidated CBD	0.8	0	-	-	-	0.8	9 (11%)	2.0	0.8	6.1
CBC	Agonist of TRPA1 receptors	0.4	0	-	-	-	0.8	0	-	-	-
CBN	Mildly psychotropic	0.8	0	-	-	-	1.6	0	-	-	-
CBG	Antagonist of CB_1_ receptors	0.4	1 (1%)	0.2	-	-	0.8	0	-	-	-
THCV	Partial agonist of CB_2_ receptors	0.4	0	-	-	-	0.8	0	-	-	-
CBDV	Homolog of CBD	0.4	0	-	-	-	0.8	0	-	-	-

**Table 3:** Comparison of early-life exposure to cannabis and tobacco.

	Prenatal Exposure to Cannabis			Childhood Exposure to Cannabis			Prenatal Exposure to Tobacco		
	Not Exposed *n* = 75	Exposed *n* = 6	*p*-Value	Not Exposed *n* = 71	Exposed *n* = 10	*p*-Value	Not exposed *n* = 72	Exposed *n* = 9	*p*-Value
Childhood exposure to cannabis									
Not exposed (*n* = 71)	67 (89%)	4 (67%)	0.10						
Exposed (*n* = 10)	8 (11%)	2 (33%)							
Prenatal exposure to tobacco ^a^									
Not exposed (*n* = 72)	69 (92%)	3 (50%)	<0.01	65 (92%)	7 (70%)	0.04			
Exposed (*n* = 9)	6 (8%)	3 (50%)		6 (8%)	3 (30%)				
Childhood exposure to tobacco ^b^									
Not exposed (*n* = 65)	62 (83%)	3 (50%)	0.05	59 (83%)	6 (60%)	0.09	62 (86%)	3 (33%)	<0.01
Exposed (*n* = 16)	13 (17%)	3 (50%)		12 (17%)	4 (40%)		10 (14%)	6 (67%)	

^a^ Prenatal exposure to tobacco was determined by the detection of cotinine in maternal urine collected at ~27 weeks gestation. The categories were as follows: exposed (cotinine > 0.05 ng/mL, the limit of detection (LOD)) and not exposed (cotinine < LOD). ^b^ Childhood exposure to tobacco was determined by the detection of cotinine in child urine collected at ~27 weeks gestation. The categories were as follows: exposed (cotinine > LOD) and not exposed (cotinine < LOD).

**Table 4:** Associations between prenatal and childhood exposure to cannabis with childhood behavior, *n* = 81.

CBCL Outcome	Prenatal Exposure to Cannabis			Childhood Exposure to Cannabis		
	Not Exposed/ Exposed	Mean Difference (95% CI)	*p*-Value	Not Exposed/ Exposed	Mean Difference (95% CI)	*p*-Value
Composite scales						
Externalizing behaviors	75/6	−2.1 (−9.8, 5.6)	0.59	71/10	3.5 (−3.4, 10.4)	0.32
Internalizing behaviors		−10.2 (−20.3, −0.2)	0.04		3.7 (−4.6, 11.9)	0.38
Total problems		−6.0 (−15.4, 3.3)	0.21		2.7 (−4.8, 10.3)	0.48
Syndrome scales						
Emotionally reactive		−3.8 (−8.2, 0.7)	0.10		4.0 (−1.1, 9.0)	0.13
Anxious/depressed		−4.5 (−10.0, 1.0)	0.11		2.5 (−1.7, 6.7)	0.24
Somatic complaints		−5.2 (−9.8, −0.6)	0.03		2.3 (−1.2, 5.9)	0.20
Withdrawn		−3.7 (−7.3, 0)	0.05		1.7 (−0.8, 4.3)	0.18
Sleep problems		−1.6 (−5.3, 2.1)	0.40		−1.3 (−4.0, 1.4)	0.33
Attention problems		−1.9 (−4.3, 0.4)	0.11		2.0 (−0.1, 4.0)	0.05
Aggressive behaviors		−1.7 (−4.1, 0.7)	0.17		3.2 (0.5, 5.9)	0.02
DSM-oriented scales						
Depressive problems		−3.3 (−7.0, 0.3)	0.08		0.3 (−2.7, 3.2)	0.87
Anxiety problems		−2.9 (−8.7, 3.0)	0.33		1.9 (−2.4, 6.1)	0.39
Attention deficit/hyperactivity		−3.3 (−9.4, 2.8)	0.29		8.0 (2.2, 13.7)	<0.01
Autism spectrum problems		−3.1 (−7.2, 0.9)	0.13		2.4 (−2.2, 6.7)	0.33
Oppositional/defiant		−2.4 (−5.8, 0.9)	0.15		3.2 (0.2, 6.3)	0.04

Abbreviations: CBCL, Child Behavior Checklist; CI, confidence interval; DSM, Diagnostic and Statistical Manual of Mental Disorders. All models adjusted for maternal age at delivery (years), maternal education (<high school, high school diploma, or some college), maternal race and ethnicity (Hispanic, non-Hispanic black, non-Hispanic white, and all other racial and ethnic groups combined), offspring sex, a non-specified maternal psychiatric diagnosis (yes or no), prenatal exposure to tobacco (urinary cotinine at 27 weeks gestation < LOD or ≥LOD), childhood exposure to tobacco (urinary cotinine at age 5 years < LOD or ≥LOD), and child age at behavioral assessment (years).

**Table 5:** Associations between prenatal and childhood exposure to cannabis with childhood cognition, *n* = 42.

	Prenatal Exposure to Cannabis			Childhood Exposure to Cannabis		
Cognitive Measure (NIH Toolbox Task)	Not Exposed/ Exposed	Mean Difference (95% CI)	*p*-Value	Not Exposed/ Exposed	Mean Difference (95% CI)	*p*-Value
Cognitive flexibility (DCCS)	37/5	2.0 (−8.7, 12.6)	0.72	38/4	−15.6 (−30.0, −1.2)	0.03
Inhibitory control (Flanker)		3.5 (−2.4, 9.4)	0.24		−9.3 (−19.1, 0.5)	0.06
Receptive language (PVT)		8.2 (−0.6, 16.8)	0.07		−9.7 (−19.2, −0.3)	0.04

Abbreviations: CI, confidence interval; DCCS, Dimensional Change Card Sort; LOD, limit of detection; PVT, Picture Vocabulary test. All models adjusted for maternal age at delivery (years), maternal education (<high school, high school diploma, or some college), maternal race and ethnicity (Hispanic, non-Hispanic black, non-Hispanic white, and all other racial and ethnic groups combined), offspring sex, a non-specified maternal psychiatric diagnosis (yes or no), prenatal exposure to tobacco (urinary cotinine at 27 weeks gestation < LOD or ≥LOD), childhood exposure to tobacco (urinary cotinine at age 5 years < LOD or ≥LOD), and child age at cognitive assessment (years).

## Data Availability

The dataset analyzed in this study is protected under an Institution Review Board protocol and is not available for distribution.

## Supplementary Materials

The following supporting information can be downloaded at: https://www.mdpi.com/article/10.3390/ijerph20064880/s1, Table S1: Characteristics of participants in the Healthy Start cohort, the pilot study, and the final analytic sample; Table S2: Fetal exposure to cannabis and offspring cognition: Scoping review of literature. Table S3: Fetal exposure to cannabis and offspring behavior: Scoping review of literature.
