# Supplementary material for: Associations between Prenatal and Postnatal Exposure to Cannabis with Cognition and Behavior at Age 5 Years: The Healthy Start Study
Source: Int J Environ Res Public Health. 2023 Mar 10;20(6):4880. doi: 10.3390/ijerph20064880 (PMC10049128; doi:10.3390/ijerph20064880)
Supplement: Supplementary file 1 [file ijerph-20-04880-s001.zip › ijerph-2223406-supplementary.pdf]

**Supplemental Table S1.** Characteristics of participants in the Healthy Start cohort, the pilot study, and the final analytic sample

| | Entire cohort (n=1,410) | Subset with cannabinoids measured in urine samples (n=199) | Analytic sample (n=81) |
|---|---|---|---|
| **Mother characteristics** | | | |
| Age (years) | 28±6 | 30±6 | 30±6 |
| Pre-pregnancy body mass index ($kg/m^2$) | 26±6 | 25±5 | 26±5 |
| Gestational weight gain (kg) | 13±7 | 13±6 | 13±6 |
| Race/Ethnicity | | | |
| Non-Hispanic White | 53% | 57% | 59% |
| Non-Hispanic Black | 15% | 11% | 4% |
| Hispanic | 25% | 25% | 31% |
| Other | 6% | 8% | 6% |
| Highest level of education | | | |
| <High school | 14% | 10% | 12% |
| High school degree | 18% | 16% | 14% |
| Some college or more | 67% | 74% | 74% |
| Household income | | | |
| <$40,000 | 29% | 25% | 22% |
| $40,001 to $70,000 | 19% | 15% | 11% |
| ≥$70,000 | 32% | 44% | 51% |
| Don't know | 20% | 15% | 16% |
| Maternal diagnosis of psychiatric illness | | | |
| Yes | 18% | 14% | 7% |
| No | 82% | 86% | 91% |
| **Child characteristics** | | | |
| Male | 52% | 49% | 48% |
| Birthweight (grams) | 3,204±537 | 3266±557 | 3319±383 |
| Gestational age (weeks) | 39±2 | 39±2 | 40±1 |
| Exclusively breastfed at age 5 months | | | |
| Yes | - | 52% | 57% |
| No | - | 49% | 42% |
| **Early-life exposure to cannabis** | | | |
| Fetal exposure to cannabis | - | | |
| Yes | - | 13% | 7% |
| No | | 87% | 93% |
| Childhood exposure to cannabis | | | |
| Yes | - | 19% | 12% |
| No | - | 81% | 88% |

**Supplemental Table S2.** Fetal exposure to cannabis and offspring cognition: Scoping review of literature

| Reference | Lead author | Study description (n) | Exposure assessment | Outcome assessment | Fetal exposure to tobacco | Other covariates | Effect |
|---|---|---|---|---|---|---|---|
| 8 | Richardson et al (1995) | USA (MHPCD), 9 months (n=520) | Self-report | Bayley Scales of Infant Development (BSID) | Covariate | Examiner, age at examination, current work/school status (other variables removed via stepwise regression) | Lower mental development scores |
| 9 | Day et al (1994) | USA (MHPCD), 18 to 36 months (n=655) | Self-report | Stanford-Binet Intelligence Scale (SBIS) | Considered as a covariate | Covariates not specified | Impaired short-term memory; Lower composite scores |
| 10 | Goldschmidt et al (2008) | USA (MHPCD), 6 years (n=648) | Self-report | SBIS | Considered as a covariate | Maternal cognitive ability, social support, race, number of people in household, Home Screen Questionnaire, Alcohol Problems of man in household, number of illnesses, maternal depression, number of siblings (final covariates determined via stepwise regression) | Impaired verbal reasoning, quantitative reasoning, and short-term memory; Lower composite scores |
| 11 | Richardson et al (2009) | USA, 3 years (n=263) | Self-report | SBIS | Considered as a covariate | Race, child sex, Home Screen Questionnaire, hostility, alcohol/tobacco/illicit drug use during pregnancy, maternal depression, number of siblings (final covariates determined via stepwise regression) | Impaired abstract/verbal reasoning; Lower composite score |
| 12 | Fried et al (2000) | Canada (OPPS), 9 to 12 years (n=146) | Self-report | Test of Visual-Perceptual Skills | Considered as a covariate | Covariates not specified | Lower scores on perceptual organization index |
| 13 | Rose-Jacobs et al (2011) | USA (BHUS) 12 to 14 years (n=137) | Biomarkers (maternal or infant urine, or meconium) and self-report | Delis–Kaplan Executive Function System | Covariate | IQ, gender, prenatal cocaine/alcohol/tobacco use, own use of cannabis/cocaine/alcohol/tobacco | Impaired design fluency total correct switching condition scores |

| | | | | | | | |
|---|---|---|---|---|---|---|---|
| 14 | Smith et al (2004) | Canada (OPPS), 18 to 22 years (n=31) | Self-report | fMRI during Go/No-Go task | Covariate | Prenatal nicotine/ alcohol/caffeine exposure and current cannabis/nicotine/alcohol considered as covariates (depending on the analysis) | Changes in neural activity during response inhibition |
| 15 | Lewis et al (2004) | USA (CWRUS), 4 years (n=374) | Detection of THC in urine immediately before or after delivery | Clinical Evaluation of Language Fundamentals—Preschool | Not included as a covariate or effect modifier | Unadjusted | Poorer scores on measure of formulating labels |
| 16 | Fried et al (2003) | Canada (OPPS), 13 to 16 years (n=31) | Self-report | Wisconsin Card Sorting Test (WCST); Wide Range Achievement Test (WRAT); Wechsler Intelligence Scale for Children (WISC); Peabody Individual Achievement Test (PIAT); Stroop test | Covariate | Covariates not specified (final covariates determined using stepwise Discriminant Function Analysis) | Lower spelling recognition scores and slower response times on a visual memory test |
| 17 | Richardson et al (2002) | USA (MHPCD), 10 years months (n=592) | Self-report | WCST; Connor's Continuous Performance Test (CPT-II); Wide Range Assessment of Memory and Learning (WRAML); Trail Making Test | Considered as a covariate | Maternal cognitive ability, social support, race, number of people in household, Home Screen Questionnaire, Alcohol Problems of man in household, number of illnesses, maternal depression, number of siblings, cocaine/tobacco/alcohol use during pregnancy (final covariates determined via stepwise regression) | Poorer performance on composite index and design memory (WRAML); More commission errors (CPT-II) |
| 18 | Singer et al (2005) | USA (CWRUS) 6 and 12 months (n=352) | THC measured in maternal or infant urine; self-report | Visual recognition memory | Covariate | Maternal age, parity, number of prenatal care visits, maternal years of education, marital status, socioeconomic status, biologic and current caregiver, PPVT/picture completion/WAIS scores, non-maternal care status, | Shorter average looking times |

| | | | | | | | |
|---|---|---|---|---|---|---|---|
| | | | | | | psychological distress and prenatal/current caregiver use of cigarette/alcohol/cannabis (final covariates determined via stepwise regression) | |
| 19 | Singer et al (2008) | USA (CWRUS), 9 years (n=371) | THC measured in maternal or infant urine; self-report | WISC; Woodcock-Johnson III Tests of Achievement | Unknown | Covariates not specified | Slower processing speed |
| 20 | Goldschmidt et al (2004) | USA (MHPCD), 10 years months (n=606) | Self-report | WRAT; PIAT | Unknown | Not clear if means presented are adjusted | Lower academic achievement |
| 21 | Goldschmidt et al (2012) | USA (MHPCD), 14 years months (n=524) | Self-report | Wechsler Individual Achievement Test (WIAT) | Not included as a covariate or effect modifier | Race, maternal education, gender, number of siblings, family income, Home Observation for Measurement of the Environment (HOME) scale; (Final covariates determined via stepwise regression) | Lower academic achievement |
| 22 | Paul et al (2021) | USA (ABCD study), 10 years, (n=11,875) | Retrospective report of cannabis use (before/after knowledge of pregnancy) | NIH Toolbox Cognition Battery | Covariate (measured before and after knowledge of pregnancy) | Maternal alcohol and tobacco use during pregnancy, maternal race and ethnicity, child sex, child age at follow up, household income, maternal education, maternal age at delivery, unplanned pregnancy, birth weight, family history of depression, mania, antisocial disorder, or anxiety, childhood alcohol or tobacco use, and singleton/multiple birth. | Lower cognition composite scores |
| 23 | Fried and Watkinson (1988) | Canada (OPPS), 1 and 2 years (n=370) | Self-report | BSID | Covariate | Covariates not specified | Higher cognition scores |
| 24 | Fried et al (1998) | Canada (OPPS), 9 to 12 years (n=131) | Self-report | WISC-III; Fluency Test; Auditory Working Memory; Tactual | Covariate | Covariates not specified (final covariates determined using stepwise Discriminant Function Analysis) | Poorer scores on WISC-III object assembly subscales |

| | | | | | | | |
|---|---|---|---|---|---|---|---|
| | | | | Performance Task; Category Test | | | |
| 25 | Willford et al (2010) | USA (MHPCD) 16 years (n=320) | Self-report | Bimanual Coordination Test | Covariate | Sex, race, maternal cognitive ability, education, marital status, social support, number of people in household, Home Screen Questionnaire, Alcohol Problems of man in household, number of illnesses, maternal depression, number of siblings, cocaine/tobacco/alcohol use during pregnancy (final covariates determined via stepwise regression) | Better performance on measure of visuomotor coordination |
| 26 | Fried and Watkinson (1990) | Canada (OPPS), 3 and 4 years (n=133) | Self-report | McCarthy's Scales of Children's Abilities (MSCA), Reynell | Considered as a covariate and effect modifier | Family income, mother's weight and pregnancy weight gain, age, education, nutrition, drug use, sex, parity, gestation, birth weight, HOME scale (final covariates not specified) | Better motor performance |
| 27 | Rose-Jacobs et al (2012) | USA (BHUS) 11 years (n=119) | THC measured in maternal/infant urine or meconium; self-report | WIAT | Covariate | Birth mothers' country of origin (US born vs. not) and years of education at recruitment; children's gender and current custody arrangement, IQ and VEX-R scores | Better academic performance; poorer verbal and memory performance |
| 28 | Singer at al (1999) | USA (CWRUS) neonates (n=74) | THC measured in maternal/infant urine or meconium; self-report | Fagan test of infant intelligence | Covariate | Covariates not specified | No association (data not presented) |
| 29 | Singer et al (2002) | USA (CWRUS) 6, 12, 24 months (n=415) | THC measured in maternal/infant urine or meconium; self-report | BSID-II | Covariate | Covariates not specified | No association (data not presented) |

| | | | | | | | |
|---|---|---|---|---|---|---|---|
| 30 | Noland et al (2003a) | USA (CWRUS) 9-12 months (n=51) | THC measured in maternal or infant urine; self-report | A-not-B task; BSID-II | Covariate | Socioeconomic status, marital status, race, maternal age, years of education, weeks of gestation, birth weight, prenatal alcohol/tobacco/cocaine use | No association (data not presented) |
| 31 | Richardson et al (2008) | USA (MHPCD) 16 months (n=261) | Self-report | BSID | Covariate | Child age, developmental stimulation, race, prenatal alcohol/tobacco/cocaine use, man in household, number of children in household, number of hospitalization, current cannabis use | No association |
| 32 | Hayes et al (1991) | Jamacia (UMJS) 4 and 5 years (n=56) | Self-report | MSCA | Covariate | | No association (data not presented) |
| 33 | O'Connell and Fried (1991) | Canada (OPPS) 6 to 9 years (n=56) | Self-report | WISC; WRAT; Stroop Test; Test of Visual Perceptual Skills; Trail making test (Part A); Developmental Test of Visual–Motor Integration | Covariate | Sociodemographic information, child's health, school progress, family size, birth order, principal language spoken at home and school, home environment, mother's personality and intelligence (final covariates determined using stepwise Discriminant Function Analysis) | No association |
| 34 | Fried et al (1992) | Canada (OPPS) 6 years (n=126) | Self-report | MSCA; Gordon delay task; Gordon vigilance task | Covariate | Covariates not specified (final covariates determined using stepwise Discriminant Function Analysis) | No association (data not presented) |
| 35 | Noland et al (2003b) | USA (CWRUS), 4 years (n=316) | THC measured in maternal or infant urine; self-report | MSCA; Motor-planning task; Tapping inhibition task; Category fluency subtest | Covariate | Maternal age, current cocaine/tobacco/alcohol use, prenatal cocaine/tobacco/alcohol use, verbal IQ | No association |
| 36 | Frank et al (2005) | USA (BHUS) 4 years (n=208) | THC measured in maternal/infant urine or meconium; self-report | Wechsler Preschool and Primary Scale of Intelligence (WPPSI) | Covariate | Prenatal cocaine, alcohol, and tobacco exposure, maternal education, birth weight, intervention at 48 months, caregiver at 48 months, caregiver's history of drug | No association |

| | | | | | | | |
|---|---|---|---|---|---|---|---|
| | | | | | | treatment, history of homelessness. | |
| 37 | Noland et al (2005) | USA (CWRUS), 4 years (n=301) | THC measured in maternal or infant urine; self-report | CPT; Peripheral Detection Task (PDT) | Not included as a covariate or effect modifier | Prenatal exposure to cocaine, caregiver current use of cannabis | No association |
| 38 | Beeghly et al (2006) | USA (BHUS) 6 and 9 years (n=160) | Self-report | Test of Language Development-Primary (TOLD-P3); Clinical Evaluation of Language Fundamentals (CELF) | Not specified | Covariates not specified | No association (data not presented) |
| 39 | Morrow et al (2006) | USA (UMSM) 7 years (n=476) | Self-report | WIAT; WISC-III | Covariate | Child sex, child age, child hearing, child education, HOME learning environmental scale, maternal age, maternal education, maternal employment, marital status, prenatal case, caregiver, caregiver employment/education, prenatal and postnatal exposure to cocaine, cannabis, tobacco, and alcohol. | Learning disabilities only among those with IQ > 70 condition |
| 40 | Mayes et al (2007) | USA (YCSC), 8 to 10 years (n=130) | Self-report | Groton maze learning test | Not specified | Not specified | No association (data not presented). |
| 41 | Bennett et al (2008) | USA (DRWJ), 4, 6, and 9 years (n=231) | Self-report | SBIS-IV | Covariate | Neonatal medical risk factors, stimulating environmental, maternal stress, maternal IQ, prenatal exposure to tobacco, alcohol, and cocaine, child age, child sex | No association |
| 42 | Carmody et al (2011) | USA (DRWJ), 6 years (n=203) | Self-report | Yale child study center attention task | Covariate | Medical complications, environmental risk, prenatal | No association |

| | | | | | | | |
|---|---|---|---|---|---|---|---|
| | | | | | | exposure to tobacco, cocaine, and alcohol, child sex. | |
| 43 | Fried et al (1997) | USA (OPPS), 9-12 years (n=131) | Self-report | WISC-III; WRAT-R; Peabody Picture Vocabulary Test (PPVT); Woodcock Reading Mastery Test; Oral cloze test; Seashore rhythm test | Covariate | Family income, maternal age, maternal and paternal education, pre-pregnancy weight, maternal drug use, child sex, the home environment, the mother's personality, the child's level of depression and anxiety, and childhood exposure to tobacco or cannabis. | No association. |
| 44 | Hurt et al (2005) | USA (CHP), 10 years (n=135) | Self-report after delivery | Gordon distractibility task | Not included as a covariate or effect modifier. | Covariates determined through backwards selection. Prenatal and postnatal exposure to cocaine, foster care, HOME score, child IQ. | No association |
| 45 | Hurt et al (2009) | USA (CHP), 12 years (n=120) | Self-report after delivery | Counting Stroop test; Go/No-go task; PPVT; Cambridge Neuropsychological Test Automated Battery (CANTAB) | Covariate | Child age, child sex, prenatal exposure to cocaine, alcohol or cocaine, HOME scores, foster care, current caregiver cocaine use, duration of cocaine exposure during pregnancy. | No association |
| 46 | Lewis et al (2010) | USA (CWRUS), 10 years (n=350) | Self-report | TOLD-I3; Comprehensive Test of Phonological Processing (CTOPP)-2 | Not included as a covariates or effect modifier | Bivariate associations. | No association |
| 47 | Day et al (2011) | USA (MHPCD) 10 years (n=580) | Self-report | SBIS | Covariate | Maternal self-report of cocaine, tobacco, and alcohol use during pregnancy, HOME scores, maternal cognitive ability, maternal depression and hostility, household income, maternal education, number of people in the household, child in maternal custody. | Not associated with learning, memory, or IQ after adjusting for covariates (data not presented). |

| | | | | | | | |
|---|---|---|---|---|---|---|---|
| 48 | Smith et al (2006) | Canada (OPPS) 18-22 years (n=31) | Self-report | fMRI during Modified n-back task; WISC; WAIS | Covariate | Prenatal nicotine, alcohol and caffeine exposure, current marijuana, alcohol and nicotine use and IQ | No association |
| 49 | Richardson et al (2015) | USA (MHPCD) 15 years (n=219) | Self-report | WISC-III | Covariate | Covariates determined by stepwise regression. Caregiver sociodemographic and psychosocial characteristics, offspring characteristics, current substance use, and prenatal substance use (cocaine, tobacco, alcohol). | No association |
| 50 | Smith et al (2016) | Canada (OPPS) 18-22 years (n=31) | Self-report | WAIS; fMRI during Modified n-back task; Go/No-go task; Counting Stroop test | Not included as a covariate or effect modifier | Current cannabis use in the offspring | No association |
| 51 | Smid et al (2021) | USA (MFMU Network), 4 years (n=1,197) | Detection of THC in urine (8-20 weeks gestation) | BSID-III | Not included as a covariate or effect modifier | Insurance type, education, race and ethnicity, and child age at examination | No association |

Study names: ABCD study, Adolescent Brain and Cognitive Development Study; BHUS Boston and Harvard Universities Study; CHP Children's Hospital of Philadelphia Study; CWRUS Case Western Reserve University Study; DRWJ, Drexel and Robert Wood Johnson Universities Study; MFMU Network, Eunice Kennedy Shriver National Institute of Child Health and Human Development Maternal-Fetal Medicine Units Network; MHPCD, Maternal Health Practices and Child Development Study; OPPS, Ottawa Prenatal Prospective Study; UMJS, University of Miami's Jamaican Study; UMSM, University of Miami School of Medicine Study; YCSC, Yale Child Study Center

**Supplemental Table S3.** Fetal exposure to cannabis and offspring behavior: Scoping review of literature

| Reference | Lead author | Study description (n) | Exposure assessment | Outcome assessment | Fetal exposure to tobacco | Other covariates | Effect |
|---|---|---|---|---|---|---|---|
| 22 | Paul et al (2021) | USA (ABCD study), 10 years, (n=11,875) | Retrospective report of cannabis use (before/after knowledge of pregnancy) | CBCL | Covariate (measured before and after knowledge of pregnancy) | Maternal alcohol and tobacco use during pregnancy, maternal race and ethnicity, child sex, child age at follow up, household income, maternal | More internalizing, externalizing, attention, thought, social, and sleep problems. |

| | | | | | | | |
|---|---|---|---|---|---|---|---|
| | | | | | | education, maternal age at delivery, unplanned pregnancy, birth weight, family history of depression, mania, antisocial disorder, or anxiety, childhood alcohol or tobacco use, and singleton/multiple birth. | |
| 51 | Smid et al (2021) | USA (MFMU Network), 4 years (n=1,197) | Detection of THC in urine (8-20 weeks gestation) | Conners' Rating Scales–Revised | Not included as a covariate or effect modifier | Insurance type, education, race and ethnicity, and child age at examination | Decreased attention scores |
| 52 | El Marroun et al (2011) | Netherlands (Generation R study), 18 months (n=4,077) | Self-report of use in first trimester | Child Behavior Checklist (CBCL) | Maternal cannabis users could also be concurrent tobacco users | Age of the child and maternal characteristics (education, ethnicity and psychopathology). | More aggressive behavior in female offspring |
| 53 | Stroud et al (2018) | USA (BAM BAM study), neonates (n=122) | Self-report | NICU Network Neurobehavioral Scale (NNNS) | Co-exposure | Varied (infant age, time since feeding, prenatal exposure to tobacco, postnatal exposure to tobacco, maternal depression, the duration of breastfeeding. | Co-exposure to cannabis and tobacco associated with decreased attention and ability to self-soothe |
| 54 | Fried, Watkinson, and Gray (1992) | Canada (OPPS), 6 years, (n=126) | Self-report | Gordan Diagnostic System | Covariate | Covariates not specified | Higher scores on impulsive/hyperactive scale and increased omission errors (reflecting decreased attention) |

| | | | | | | | |
|---|---|---|---|---|---|---|---|
| 55 | Goldschimdt et al (2000) | USA (MHPCD), 10 years (n=636) | Self-report in each trimester | CBCL, Swanson, Noland, and Pelham (SNAP), Teacher's Report Form (TRP) | Covariate | Maternal education, race, marital status, work/school, family income, maternal depression/hostility, alcohol and tobacco use during pregnancy | Increased hyperactivity, impulsivity, and inattention (SNAP); increased delinquency (CBCL); Increased delinquency and externalizing problems (CBCL). |
| 56 | Gray et al (2000) | USA (MHPCD), 10 years (n=636) | Self-report in each trimester | Children's Depression Inventory (CDI) | Not included as a covariate or effect modifier | Varied (childhood IQ, maternal tobacco use at age 10 years, childhood hospitalizations, HOME score, life events) | More depressive symptoms. |
| 57 | Leech et al (2006) | USA (MHPCD), 10 years (n=636) | Self-report | CDI, Revised Children's Manifest Anxiety Scale (RCMAS) | Covariate | Attention problems, household density, prenatal, injuries, and marijuana exposure. | Higher combined depression and anxiety score |
| 58 | Godleski et al (2018) | USA, 24 and 36 months (n=247) | Self-report in each trimester and biologically verified (maternal saliva and infant meconium) | Brief Infant Toddler Social Emotional Assessment (BITSEA) | Effect modifier | Demographic risk and child sex. Mediators included breastfeeding duration, maternal warmth/sensitivity and maternal affective dysregulation | No association |
| 59 | Larkby et al (2011) | USA, 16 years (n=592) | Self-report in each trimester | DSM-IV Diagnosis of Conduct Disorder | Covariate | Prenatal exposure to tobacco, marijuana, cocaine (& other illicit drugs), income, race, gender, parenting style, life events, home environment, | No association |

| | | | | | | | |
|---|---|---|---|---|---|---|---|
| | | | | | | family history of alcohol, and maternal psychopathy | |
| 60 | Leech et al (1999) | USA (MHPCD), 6 years (n=636) | Self-report in the first trimester | Continuous Performance Task (CPT) | Covariate | Child characteristics, environmental characteristics, maternal characteristics, current maternal substance use, prenatal substance exposure | Second trimester cannabis use was associated with **more** errors of commission (reflecting impulsivity) and **fewer** omission errors (reflecting improved attention) |
| 23 | Fried and Watkinson (1988) | Canada (OPPS), 1 and 2 years (n= 370) | Self-report | Infant Behavior Record (IBR) | Covariate | Covariates not specified | No association |
| 24 | Fried et al (1998) | Canada (OPPS), 9 to 12 years (n=131) | Self-report | Gordon Vigilance Test | Covariate | Covariates not specified (final covariates determined using stepwise Discriminant Function Analysis) | Improved sustained attention (more correct responses and fewer commission errors) |
| 61 | Richardson et al (1989) | USA (MHPCD), neonates, (n= 373) | Self-report in each trimester | Neonatal Behavior Assessment Scale (NBAS) | Covariate | Birth weight, other substance use, infant age at exam, NBAS examiner | No association |
| 62 | de Moraes Barros et al (2006) | Brazil, neonates (n=561) | Cannabinoids measured in maternal hair and neonatal meconium analysis | NICU Network Neurobehavioral Scale (NNNS) | Excluded | Sex, gestational age at birth, postnatal age, and the product of gestational x postnatal age | Altered neurological performance at birth |
| 63 | Murnan et al (2021) | USA, 3.5 years, (n=63) | Self-report or detection of THC in maternal urine | Toy Behind Barrier task; Bobo Interaction task; Leifer-Roberts Response | Covariate | Child race, sex, prenatal tobacco exposure, household income, caregiver marital status, and | Associated with more aggression (Bobo task, CBCL), withdrawn symptoms, externalizing behaviors, and |

| | | | | | | | |
|---|---|---|---|---|---|---|---|
| | | | | Hierarchy Questionnaire (LRRHQ); CBCL | | caregiver executive functioning | oppositional defiant behaviors. |
| 64 | Hunter et al (2021) | USA, 4 years (n=83) | Self-report | CBCL | Not included as a covariate or effect modifier | Covariates not specified | More attention problems, sleep problems, social withdrawn problems, anxiety/depression, emotionally reactive, somatic complaints, aggressive behavior, and total problems. |
| 65 | Cioffredi et al (2022) | USA (ABCD), 9-10 years (n=672) | Retrospective report of cannabis use (before/after knowledge of pregnancy) | CBCL | Matched | Parental total problems, maternal age, months breastfed, prematurity, race and highest household education. | More externalizing behaviors, total problems, attention problems, and thought problems. |
| 66 | DiGuiseppi et al (2022) | USA (SEED); 30-68 months (n=4,254) | Self-report | Diagnosis of Autism Spectrum Disorder | Covariate | SEED phase, child sex, maternal race/ethnicity, maternal education, and peri-pregnancy use of tobacco, alcohol, and other illicit drugs. | No association |

Study names: ABCD study, Adolescent Brain and Cognitive Development Study; MFMU Network, Eunice Kennedy Shriver National Institute of Child Health and Human Development Maternal-Fetal Medicine Units Network; MHPCD, Maternal Health Practices and Child Development Study; OPPS, Ottawa Prenatal Prospective Study; SEED, Study to Explore Early Development
